# Secret Remedies and Branded Products

**Published:** 1908-01-04

**Authors:** 


### Therapeutics.

### SECRET REMEDIES AND BRANDED PRODUCTS.

In this age of competition, given an open market, the best article commands the largest sale, which, in pursuance of the law of the survival of the fittest, is only right and natural. The competitive market is therefore a place to be shunned by business men who are not able to give value for money; and so it is that when competition is keenest those who are not able to face it circumvent it. In a fair and open market the price of a man's property is fixed by his competitor; its value can be assessed to a nicety. In a market where there is no competition the value of an article offered is fixed by the owner, the cost of production being an insignificant factor in determination of price. Such a market is naturally sought by the astute merchant who has been unable to hold his own in the open field, and hence the enormous and continually increasing supply of branded products, which are foisted on the public at artificially enhanced prices to the detriment of the sale of genuine products whose value is fixed on a real business basis. Competition hard driven thus encourages monopoly, and this is one of the reasons why we are passing through an era of fancy titles and branded goods. Advertisers have by their energy and astuteness so created the fashion that nowadays people must have something with a name, whether it be pickles, or pills, or even bread.

Under these circumstances it is not surprising that the prevailing fashion has assailed the medical profession. The practitioner of medicine has, in fact, succumbed before the invasion of the proprietary product to an extent which has become alarming, and threatens danger to his practice and to the health of the public. During the last 20 or 25 years the whole aspect of the art of prescribing has undergone a complete change; indeed, so great has been the change that it is now hardly recognisable as an art. Standard preparations and pharmacopœial products are being neglected, while their place is being usurped by ready-made proprietary products. Sometimes these preparations are of secret composition, sometimes the character of the ingredients is stated, and sometimes the exact proportions of each constituent are divulged, although the bare statement of the makers is not always to be relied upon. But all three varieties are the property of private firms in business solely to make money. The standard of proprietary products is regulated by the whim of the owners, sometimes altered to meet the requirements of the law or market advances, whereas on the other hand, the standard of pharmacopœial products is fixed, and any deviation from that standard entails an offence punishable by law.

It would hardly be possible to find a member of the medical profession who would not recoil with disgust from the daring and impudent assurance of the "quack" medicine vendor; and yet when the matter is closely studied it will be found that the proprietors of many of the branded goods advertised to physicians by means of travelling touts and otherwise are as extravagant, and often as unscrupulous, as their less refined confrères, the makers of 1s. 1½d. "cure-alls." It will be the purpose of a series of articles which will appear in this section to show the danger underlying this departure from scientific methods of prescribing.

To return to the question of secret remedies—whether those sold to a credulous public or through the prescription of a tolerant physician—there is no logical reason for their existence. Science abhors secrecy, its constant aim being to lay bare everything. Unscrupulous traders, on the other hand, cling to secrecy, because they have something to hide: the moment their hidden secret is revealed they are, as a rule, exposed as swindlers. Curiously enough, the law is very generous to owners of so-called secrets where a medicine for human use is concerned, and is only inquisitive and anxious where the lower animals are affected. Thus by the "Sheep Scab Order of 1905" it is provided that manufacturers of sheep dips shall, before placing their compounds on the market, obtain the sanction of the Board of Agriculture and Fisheries. This Order, which was issued in pursuance of the powers vested in the Board by the Diseases of Animals Act, 1894 to 1903, requires that the composition of the dips shall be disclosed before sanction for their sale is granted. The Fertilisers and Feeding-stuffs Act, 1906, requires makers of artificial fertilisers for the soil and makers of artificially prepared food for cattle or poultry to provide the purchaser with an invoice giving a correct statement of the percentages of the various ingredients. The law thus protects sheep, the soil, horses, cows, and chickens against the impostures of the advertising quack, but is practically powerless against the manufacturer of fraudulent medicines and patent foods for human use. The result of the disclosure of the composition of garden manures has been to show that many of the much-vaunted products sold at fictitious prices are practically worthless. The result of the private analysis of many secret remedies goes to show that those which are not positively harmful are usually without value. The law protects sheep, but is quite callous about human life. However, the position is clearly illogical, and the same legislature which looks after the welfare of the chicken cannot continue to disregard the health of the human infant.

In most civilised countries, except our own, the authorities have already placed restrictions on the sale of nostrums, and in our own colonies the net is gradually being drawn over the exploiters of worthless "secrets." The Commonwealth of Australia adopted the wise plan of appointing a Royal Commission of Inquiry into Secret Drugs, and the result of the inquiry has been made known in the form of a Government publication, to the discredit of many preparations enjoying a vast sale. Up to the present our own Government has adopted a singularly indifferent attitude with regard to suggested reforms affecting the advertising and sale of secret proprietaries.

